# E6/E7 oncogenes in epithelial suprabasal layers and estradiol promote cervical growth and ear regeneration

**DOI:** 10.1038/oncsis.2017.73

**Published:** 2017-08-28

**Authors:** C García, D Hernández-García, C Valencia, V Rojo-León, J-R Pérez-Estrada, M Werner, L Covarrubias

**Affiliations:** 1 Instituto de Biotecnología, Universidad Nacional Autónoma de México. Av. Universidad 2001, Cuernavaca, Morelos, México

## Abstract

Tissue growth is a common characteristic of carcinogenesis and regeneration. Here we show that suprabasal expression of human papillomavirus (HPV)16 E6/E7 oncogenes in Tg(K6b-E6/E7) mice, similar to that observed in HPV-infected human tissue, and estradiol increased cervical epithelium growth and ear-hole closure efficiency. Oncogenes in combination with estradiol had a significant contribution to the proliferation of suprabasal cells of cervical epithelium that correlated with an increased expression of keratin genes. Remarkably, long-term treatments with estradiol resulted in evident cellular and tissue abnormalities indicative of a precancerous phenotype. Regenerating ear epithelium of transgenic mice also showed increased suprabasal cell proliferation and expression of keratin genes. Unexpectedly, we observed higher ear regeneration efficiency in adult than in young female mice, which was further increased by E6/E7 oncogenes. Supporting a role of estradiol in this phenomenon, ovariectomy and treatment with an estrogen receptor inhibitor caused a significant reduction in regenerative capacity. Our data suggest that Tg(K6b-E6/E7) mice are unique to mimic the initial stages of HPV-mediated cervical carcinogenesis, and ear regeneration could facilitate the elucidation of mechanisms involved.

## Introduction

Growth is a condition that predisposes tissues to initiate a carcinogenic process.^1, 2^ Accordingly, tissues that carry out continuous renewal during adult life have high incidence of tumors,^2^ and their specific stem cells are the main candidates for cancer initiation. In addition, injury, another condition associated with cancer development,^3^ forces tissues to recruit cells from other niches to fulfill the high demand of cells needed for healing.^4^ The self-renewal capacity of stem cells places them as the major target for the accumulation of mutations that trigger a carcinogenic process.^2^ However, recent studies have shown the emergence of tumors from transient proliferating progenitors or from postmitotic differentiated cells.^5, 6^

A hallmark of tissue renewal, repair and regeneration is the activation of cell proliferation, differentiation and growth.^7^ Thus, it is not surprising that proto-oncogenes and tumor suppressors participate in these processes by promoting cell proliferation and differentiation, respectively.^8^ In this context, it is interesting the evolution of the Ink4a locus encoding in mammals for Arf and p16Ink4a, key regulators of the p53 and pRb1 pathways; this locus in lower vertebrates, including amphibians, encodes exclusively for p16Ink4a, possibly causing a marked difference in cell cycle regulation.^8, 9^ Furthermore, in addition to the increase in cell proliferation, reducing the activity of these tumor suppressors facilitates cell reprogramming/dedifferentiation,^10, 11^ a process common to regeneration and cancer.

Cervical–uterine cancer is the cause of many deaths worldwide. Cervical–uterine endometrium is a continuously renewing tissue during the whole reproductive life. The estrous cycle is a hormonally regulated process whose phases can be distinguished by the marked growth changes occurring in the reproductive tract.^12^ Slow dividing stem cells have been identified in this tissue, which proliferate upon estrogen receptor (ER) stimulation.^13, 14^ Infection by human papillomavirus (HPV) is a major etiological factor associated with cervical–uterine cancer, though cancer incidence in infected women is low.^15^ Although, it is predicted that stem cells are the target of HPV oncogenes during cancer initiation, viral replication and expression of major HPV oncogenes (e.g., E6, E7) occur in the suprabasal layers of the growing epithelium to ensure a productive infection.^16^ Accordingly, *LacZ* expression driven by the Long-Control-Region of HPV was located in suprabasal layers.^17^ Actually, it has been proposed that E6/E7 could promote cell cycle re-entry in upper epithelial layers.^16, 18^

Mouse models of cervical–uterine cancer have been developed previously by directing the expression of HPV oncogenes to the basal layer of epithelia (K14+ cells).^19, 20^ Here, we determined the effect of E6/E7 HPV oncogenes on cervical epithelium renewal when expressed in suprabasal layers (K6b+ cells) during the growing phases. In addition, taking advantage of the increased regenerative capacity of these mice,^21^ we compared the growth effect on ear regeneration and cervix renewal in response to oncogenes and/or estradiol.

## Results

### Growth induction without changing the estrous cycle-associated pattern in Tg(K6b-E6/E7) cervix

**Figure 1:**
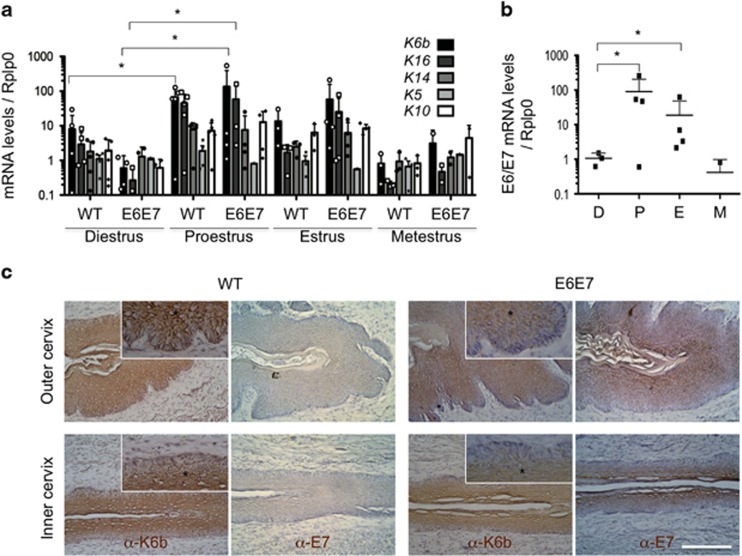
Keratin genes and *E6/E7* expression in the cervix during the estrous cycle. Cervix samples were taken from 9-week-old wild type (WT; *n*=4) or Tg(K6b-E6/E7) (E6E7; *n*=4) mice at specific estrous cycle phases (D, diestrus, E, estrus; P, proestrus and M: metestrus) and keratin genes (**a**) or *E6/E7* (**b**) mRNA levels determined by RT-qPCR (see Materials and Methods). Observe the highest expression levels during the growing phases (P and E). Immunolocalization of K6b and E7 in the epithelium of outer and inner cervix at proestrus showed the presence of these proteins in suprabasal layers (* mark), with a more abundant E7 presence in the most suprabasal layers (**c**). *P<0.05; scale bar, 100 μm.

Keratin gene expression in the cervix of wild-type (WT) mice significantly changed during the estrous cycle (Figure 1a). Except for *K5*, the genes encoding all keratins tested increased during the diestrus–proestrus transition. *K14* and *K10* expression increased about fivefold, whereas *K6b* and *K16* expression increased between 10- and 20-fold; the lowest mRNA levels were reached at metestrus. Similar pattern was observed in Tg(K6b-E6/E7) mice but upregulation was much higher than that determined in WT mice reaching up to 100-fold for *K6b* and *K16* and 5- to 10-fold for *K14* and *K10* (Figure 1a); in the cervix of Tg(K6b-E6/E7) mice, mRNA levels at metestrus were higher than those in equivalent samples of WT mice. As expected, *E6/E7* expression correlated with the pattern observed for *K6b* (Figure 1b).

**Figure 2:**
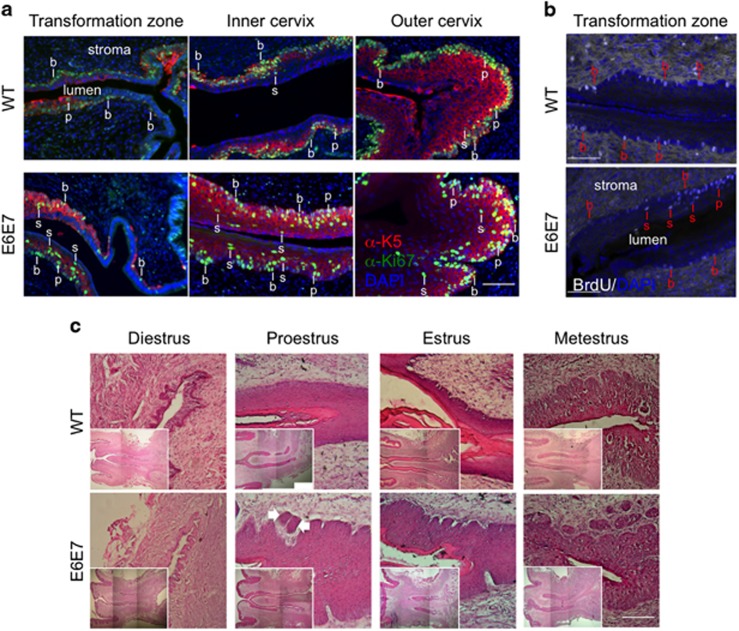
Growth pattern of WT and Tg(K6b-E6/E7) cervix during the estrous cycle. Ki67 determination (**a**) and 2-h BrdU incorporation (**b**) during the growth phases in transformation zone, and inner and outer cervix showed that oncogenes added suprabasal proliferating cells to those regularly present in the basal layer. Note the basal to suprabasal K5 distribution gradient, particularly evident in the outer cervix. Some basal (b), parabasal (p) and suprabasal (s) cells are indicated. (**c**) A histological comparison between WT and Tg(K6b-E6/E7) cervix of 9-week-old mice showed similar tissue appearance at each estrous cycle phase but a thicker epithelium during the growing phases in the latter than in the former, in some cases evidenced as epithelia invading the stroma (arrows). Scale bar, 100 μm.

The *E6/E7* expression pattern along the estrous cycle and distribution of E7 protein in the cervix at proestrus was similar to that of K6b and restricted to suprabasal layers in either outer or inner cervix (Figure 1c); oncogene proteins were more abundant in the most suprabasal layers. Distribution of K5, the K14 companion keratin usually located in the basal layer, was not altered by oncogenes in the outer cervix, where it showed an evident basal–suprabasal gradient (Figure 2a). However, this gradient was only detected in the thicker than WT transformation zone and inner cervix epithelia of Tg(K6b-E6/E7) mice (Figure 2a).

**Figure 3:**
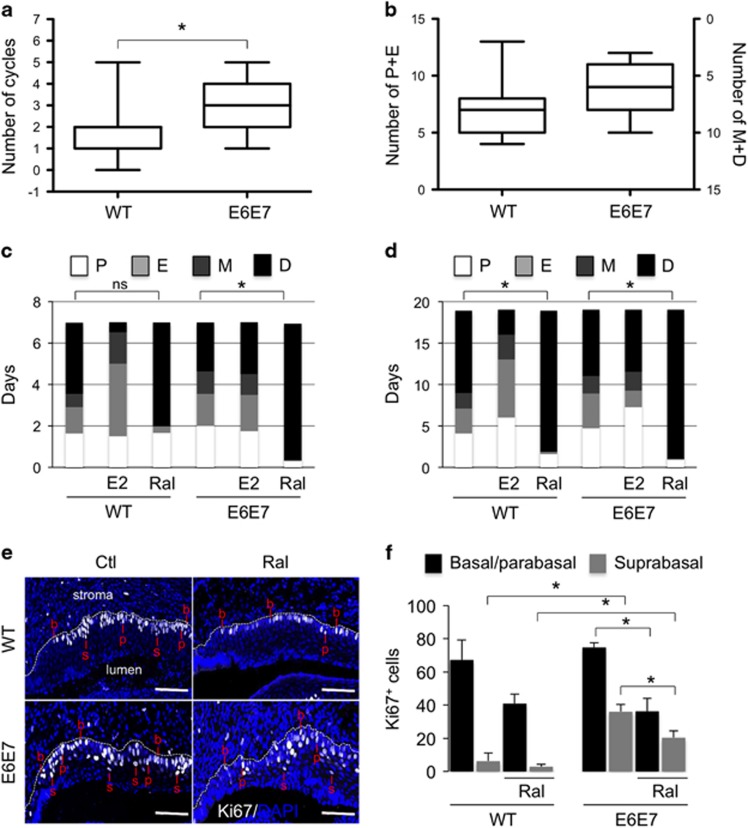
Cooperation between E6/E7 and estradiol during the estrous cycle. Frequency of P–E sequence (**a**), number of days detected in P or E and in M or D (**b**) determined along 19 days. Note that the frequency of P–E sequence, and apparently the number of days in the growing phases, was higher in Tg(K6b-E6/E7) (*n*=11) than in WT (*n*=11) mice. WT (*n*=2) but not Tg(K6b-E6/E7) (*n*=4) mice continuously treated with E2 were more days at proestrus–estrus (P–E) during a 7 (**c**) or 19 days (**d**) period, whereas raloxifene (Ral) blocked the estrous cycle of both WT (*n*=3) and Tg(K6b-E6/E7) (*n*=3) mice. (**e**, **f**) Ki67 immunopositive (Ki67+) cells determined in the cervix of mice at proestrus treated for 14 h with Ral (or PBS); note that the increase in the number of Ki67+ cells in Tg(K6b-E6/E7) mice was restricted to suprabasal layers and the reduction in these cells was observed in both WT (*n*=3) and Tg(K6b-E6/E7) (*n*=4) mice. Some basal (b), parabasal (p) and suprabasal (s) cells are indicated. *P<0.05; NS, not significant. Scale bars, 100 μm.

In agreement with the expected effect and expression pattern of HPV oncogenes in the cervix of Tg(K6b-E6/E7) mice, the increase in proliferating cells was restricted to suprabasal layers (Figures 2a and b; see also Figures 3e and f). Actually no Ki67+ cell was found in the suprabasal layer of WT mice and the few BrdU+ cells found (1 for every 10 basal) could be recently differentiated cells. These observations correlated with a higher growth of cervical epithelium of Tg(K6b-E6/E7) in comparison with that of WT mice, evident during proestrus, where epithelial protrusions into the stroma were noted (Figure 2c). Despite these abnormalities, the epithelium of cervix established the major characteristics of each estrous phase (Figure 2c).

### Increased number of cycles and longer proestrus–estrus phase in Tg(K6b-E6/E7) mice

In order to obtain indications of estrous cycle progression, we compared Tg(K6b-E6/E7) and WT mice regarding the number of cycles, as determined by the frequency of sequential proestrus–estrus phases, and the time staying at the growth phases, as determined by the frequency of proestrus and estrus, both within a defined number of days (19 days). Notably, the frequency of cycles (Figure 3a) and, apparently, the incidence of growth phases (Figures 3b–d) were higher in Tg(K6b-E6/E7) than in WT mice. Nonetheless, the initiation of each cycle was still dependent on estradiol as cycling was blocked in both Tg(K6b-E6/E7) and WT mice treated with raloxifene (Figures 3c and d).

Cytological analysis after a short-term β-estradiol (E2) treatment showed a tendency of mice to stay in the growth phases of the estrous cycle (Figures 3c and d). This result suggests a possible effect of estradiol on epithelium during the growth phases. To test this hypothesis, mice were treated with raloxifene once proestrus had initiated and, then, cell proliferation evaluated by the presence of Ki67 (Figure 3e). Despite the higher number of suprabasal proliferating cells due to oncogene expression, reduction in the number Ki67+ cells was observed in both WT and Tg(K6b-E6/E7) mice with a similar effect in basal/parabasal and suprabasal cells (Figure 3f); no indication of cell death was detected in the presence of raloxifene (Supplementary Figure S1). These results confirm the critical role of estradiol in epithelial growth and support the cooperation with oncogenes during the proestrus–estrus phase.

**Figure 4:**
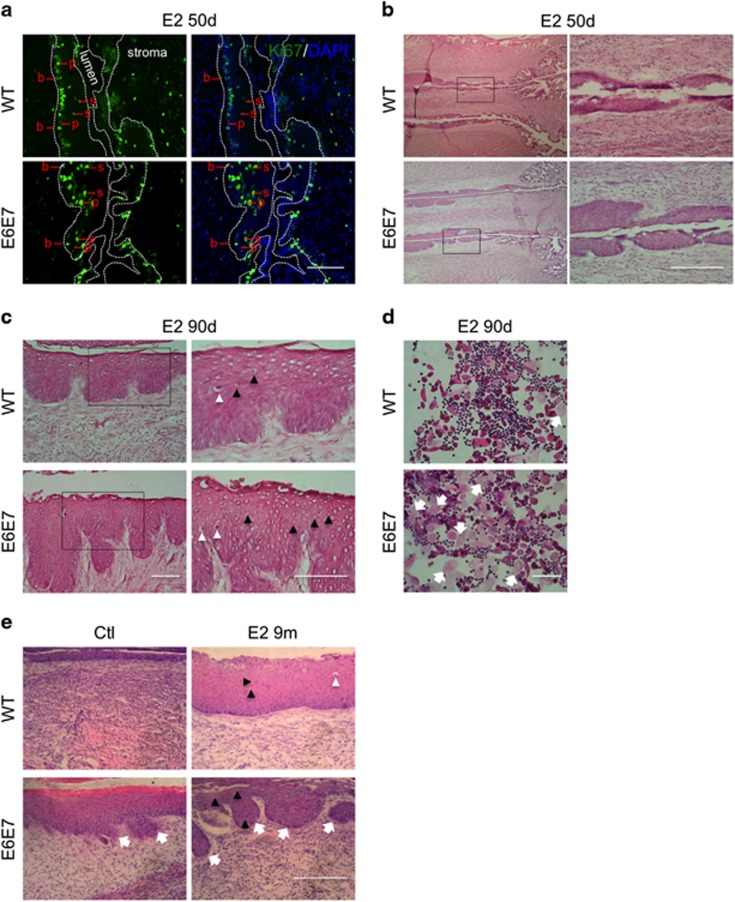
Cooperation between E6/E7 and estradiol in the induction of an epithelial cancerous phenotype. (**a**) Ki67 immunopositive (Ki67+) cells determined in transformation zone of WT (*n*=3) and Tg(K6b-E6/E7 (*n*=4) mice treated with E2 for 50 days; note the emergence of Ki67 cells in suprabasal layers of cervical epithelium of WT mice and an increased number of these in Tg(K6b-E6/E7) mice (50 days). Some basal (b), parabasal (p) and suprabasal (s) cells are indicated. (**b**) Thickening of the cervical epithelium of mice treated with E2 for 50 days. Note the epithelial hyperplasia in the transformation zone, particularly evident in that of Tg(K6b-E6/E7 mice. (**c**) Koilocyte-like cells detected in mice treated with E2 for 90 days. Koilocytes-like cells, identified by a large clear cytoplasm (black arrowheads), were found in samples from mice treated with E2 and in a larger number in samples from Tg(K6b-E6/E7) mice; sometimes, leukocyte infiltration was detected in these samples (white arrowheads). (**d**) Bi-nucleated cells (arrows) in Tg(K6b-E6/E7) vaginal smears. (**e**) Hyperplasia was the main outcome in cervix of WT animals at 9 months of E2 treatment, whereas in Tg(K6b-E6/E7) signs of dyspasia and carcinoma *in situ* were present (arrows). Scale bars, 100 μm.

In contrast with short-term E2 treatments, proliferating cells in suprabasal layers of WT cervical epithelia were detected (about 28% of total Ki67+ cells) after long-term treatments (>1.5 months) and, as expected, many more of those were detected (about 44% of total Ki67+ cells) in the cervical epithelia of Tg(K6b-E6/E7) mice (Figure 4a). Accordingly, long-term E2 treatments thickened the epithelia of the transformation zone of WT cervix (*n*=4), though a more pronounced growth in this region occurred in Tg(K6b-E6/E7) mice (*n*=3; Figure 4b). Interestingly, as a possible indication of a precancerous condition, koilocyte-like cells (i.e., squamous cells with a clear cytoplasm^22^) were found in the grown cervical epithelia of both WT and Tg(K6b-E6/E7) mice, with an evident increased number in the latter (Figure 4c). In addition, a high frequency of bi-nucleated cells was found in vaginal smears of E2-treated Tg(K6b-E6/E7) mice (Figure 4d). In WT mice, even after 9 months of E2 treatment, hyperplasia was the major phenotype of cervical epithelium. In contrast, in Tg(K6b-E6/E7) mice, hyperplasia and an irregular epithelium resembling a dysplasic tissue were frequently observed even in the absence of the hormone, as it was described above, but E2 treatment notoriously exacerbated the hyperplastic phenotype showing deep dysplasic protrusions and epithelial islands that commonly characterize the carcinoma *in situ* (Figures 4c and e).

### Ear regeneration in adult mice is influenced by estradiol and E6/E7 oncogenes

**Figure 5:**
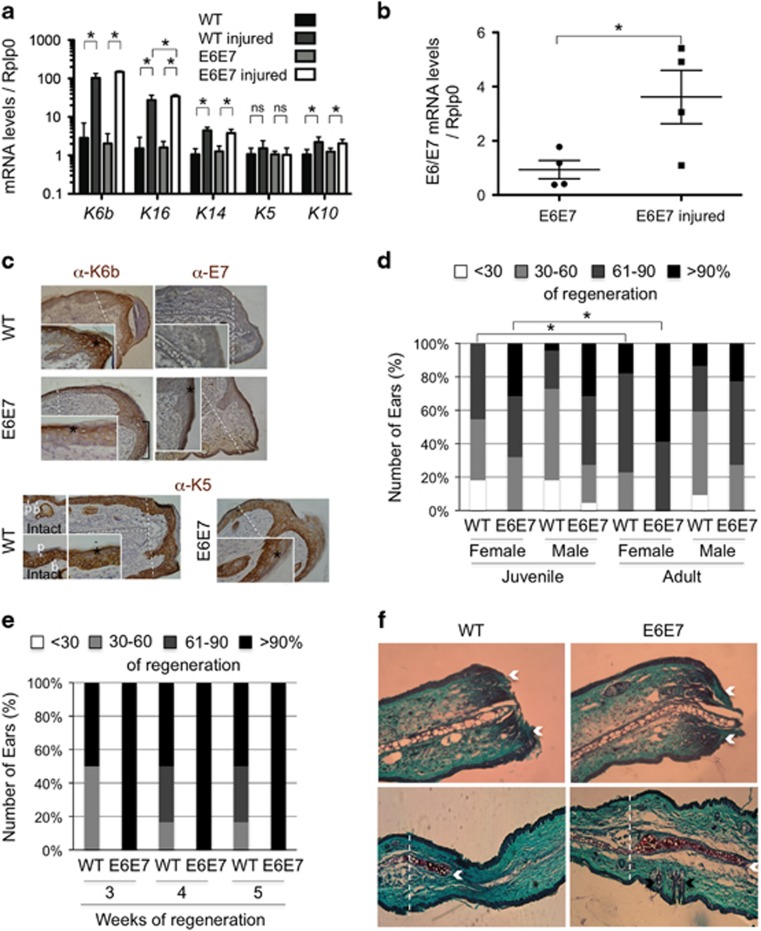
Keratin genes and *E6/E7* expression during ear regeneration in adult mice. Keratins (**a**) and E6/E7 (**b**) mRNA levels were determined in samples of 3-month-old mice from intact ears or from those perforated 7 days earlier (WT, *n*=7; E6E7, *n*=5); mRNA levels were higher in samples from perforated than from intact ears. (**c**) Immunolocalization of K6b and E7 in the epidermis of regenerating ears showed these proteins in suprabasal layers around the wound borders (* mark), but K6b was also in the basal area of the growing epidermis and K5 more restricted to basal layers in the intact tissue. (**d**) Regeneration efficiency of holes in ears of juvenile (6-weeks-old) and adult (3-months-old) (WT, *n*=22; E6E7, *n*=22); ear regeneration was more efficient in adult than in juvenile female mice, and even better in Tg(K6b-E6/E7) mice, whereas no difference due to age was detected in males. (**e**) Evaluation of hole closure at 3, 4 and 5 weeks after perforation (*n*=6) indicated that higher efficiency was related to a faster regeneration process. (**f**) Sections of ears of WT and Tg(K6b-E6/E7) mice 24 h (upper panel) or 3 months after perforation (lower panel) stained with fast-green and safranin to visualize the re-epithelization process (white arrows indicate the border of the epithelial tongue covering the wound), the emergence of new cartilage (white arrows) and the formation of new hair follicles (black arrows); the dashed line indicates the site of injury. Note that epidermis of intact ears is very thin, normally composed of few suprabasal layers. NS, not significant; b, basal; p, parabasal cells. *P<0.05; scale bar, 100 μm.

Induction of *K6b*, *K16*, *K14, K5* and *K10* expression during epidermal growth in regenerating as compared with intact ears was similar to that observed in the cervix during the diestrus–proestrus transition (Figure 5a). Induction was higher for *K6b* and *K16* (around 20- to 50-fold) than for *K14, K5* and *K10* (2- to 5-fold). As in the cervix, E6/E7 oncogenes promote growth of epidermal tissue in regenerating ears,^21^ but induction of keratin gene expression remained in the same range as in regenerating WT ears (Figure 5a) and that of *E6/E7* expression followed similar pattern (fourfold; Figure 5b). Distribution pattern of E7 protein during regeneration was found very similar to that of K6b (Figure 5c). Around the wound borders (dashed line), restriction to suprabasal layers was evident; similar pattern was observed in the growing area where the basal layer was well defined (square bracket) and E7 was better detected in the most upper layers (Figure 5c); in disorganized epidermis, commonly seen in the regenerating area, this restriction was not evident. K5, in contrast with the basal restriction in intact epidermis, was widely distributed in the growing area with a basal to suprabasal gradient pattern in both WT and Tg(K6b-E6/E7) mice (Figure 5c).

Regeneration is expected to be more efficient in young animals; accordingly, most ear regeneration studies have been done with mice younger than 2 months. Therefore, it was unexpected to find that the hole in ears of female WT adult mice regenerated more efficiently than in those of young female animals (Figure 5d). A more pronounced effect was noted in ears of Tg(K6b-E6/E7) adult mice (Figure 4d). Regeneration efficiency was related with faster hole closure (Figure 5e). As previously demonstrated for young animals,^21^ closing of ear holes involved fast re-ephitelization and, as expected for regeneration rather than a wound repair with scar, formation of new cartilage and hair follicles was observed (Figure 5f).

**Figure 6:**
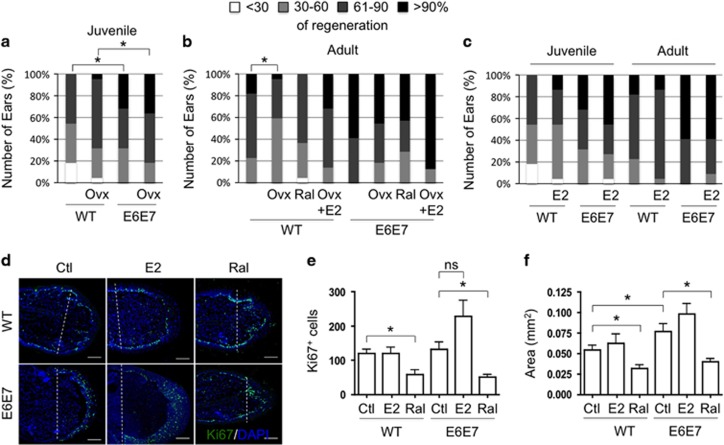
Cooperation between E6/E7 oncogenes and estradiol in ear regeneration. Ear regeneration in 6-week-old (juvenile; **a**) and 3-month-old (adult; **b**) WT and Tg(K6b-E6/E7) mice after ovariectomy (Ovx; *n*=44) or treatment with raloxifene (Ral; *n*=22); some ovariectomized mice were also treated with E2 (Ovx+E2; *n*=22). Note that the reduction in estradiol levels (Ovx, Ral) decreased regeneration efficiency, whereas E2 addition recovered it. (**c**) Exogenous E2 had no effect in juvenile (*n*=22) or adult (*n*=22) female mice. (**d**, **e**) The distribution and number of Ki67+ cells were determined 10 days after punching in regenerating ears of juvenile WT and Tg(K6b-E6/E7) mice that were treated with raloxifene (Ral; *n*=3) or with PBS (Ctl; *n*=3). A marked reduction in Ki67+ cells was observed in samples from mice treated with raloxifene; in the case of Tg(K6b-E6/E7) mice, the reduction was mostly restricted to suprabasal cells. (**f**) The total growing area of epidermis was delimited and measured from the initial injury site (dashed line); note that the growth pattern after treatment with E2 or raloxifene is similar to that of proliferating cells in the epidermis. NS, not significant; *P<0.05.

Hole closure efficiency decreased in juvenile ovariectomized mice (Figure 6a); this effect on regeneration was evident in adult WT mice but not noted in Tg(K6b-E6/E7) mice (Figure 6b). Supporting estradiol as the relevant molecule removed by ovariectomy, the exogenous addition of E2 fully recovered the regeneration capacity of WT ovariectomized mice (Figure 6b). In addition, raloxifene produced the same effect as ovariectomy especially noted in Tg(K6b-E6/E7) (Figure 6b). Addition of E2 to WT or Tg(K6b-E6/E7) mice had no effect on ear hole closure (Figure 6c). However, exogenous E2 in males did produce a detectable improvement in regeneration (Supplementary Figure S2). This observation is relevant considering that an inhibitory factor appeared present in males, as demonstrated by the increased ear regeneration capacity of castrated males (Supplementary Figure S2).

### Estradiol promotes cell proliferation during ear regeneration

In young mice, E6/E7 improved regeneration capacity in association with increased cell proliferation.^21^ Accordingly, the number of proliferating cells increased in both WT and Tg(K6b-E6/E7) adult mice in the regenerating area but the latter, likely due to oncogene expression, showed a higher proportion of proliferating cells in suprabasal than in basal layers (Figure 6d). In agreement with a poor effect of exogenous E2 on regeneration, the change in the number of proliferating cells (Ki67+ cells) in regenerating ears of mice treated with E2 was not significant (Figure 6e). However, treatment with raloxifene caused a reduction in the number of proliferating cells in both WT and Tg(K6b-E6/E7) mice (Figure 6e). BrdU incorporation supports this conclusion and, in addition, the apparent accumulation of BrdU+ in the wound borders suggests that estradiol may also contribute to the important migration at early stages of regeneration (Supplementary Figure S3).^21^ Therefore, E6/E7 cooperate with estradiol to promote proliferation, and possibly also migration, of epidermal cells during regeneration. The number of proliferating cells determined by estradiol and/or oncogenes is the likely factor responsible of epidermal growth, since direct measurement of epidermal tissue in the growing area followed the same pattern (Figure 6f). The contribution of proliferating hair follicle cells to the regenerating area was observed in few instances (Supplementary Figure S4), but could not be considered a major source of cells for ear regeneration.

## Discussion

### Contribution of suprabasal epithelial cells to carcinogenesis and regeneration

In stratified epithelia, the proliferative compartment is restricted to the basal layer. Here, cycling stem cells and progenitors live together and derive into the non-dividing terminally differentiated cells of suprabasal layers which mature up to producing the cornified tissue.^13, 23^ Commonly, hyperplasia and dysplasia in skin and cervix are characterized by a high number of proliferating cells located in suprabasal layers.^19, 20, 24^ The recurrent hypothesis to this phenotype is the invasion of basal proliferating cells into upper layers. However, recent data^5, 6^ open alternative hypotheses that need to be considered to trace the origin of cancer-initiating cells.

It has been proposed that a productive cycle of HPV in the cervix starts with the infection of basal layer cells.^16^ Upon differentiation, suprabasal cells start to express the early viral genes.^16^ Therefore, the primary cells for E6/E7 action are suprabasal cells which, in response, re-enter into the cell cycle. Tg(K6b-E6/E7) mice reproduced this pattern in the cervix, and accordingly, cell proliferation was promoted in suprabasal layers. This function is active in the growing phases of the estrous cycle when *K6b* was detected to be highly expressed. Since suprabasal *E6/E7* expression did not affect the number of proliferating basal cells, few of these cells contributed to the suprabasal proliferation pattern observed. Therefore, suprabasal cells are relevant candidates to carry the role of cancer-initiating cells in the cervix infected with HPV. In contrast, although Tg(K14-E6/E7) mice can reproduce many aspects of cervical–uterine cancer when chronically treated with E2,^19, 20^ the compelled premise for the search of cancer-initiating cells in these mice is that they are stem/progenitor cells of the basal layer. Therefore, these mice unlikely model the initiation of cervical carcinogenesis in humans infected with HPV as, possibly, Tg(K6b-E6/E7) mice do.

It is interesting that, in contrast with Tg(K14-E6/E7) mice,^25^ the interfollicular epidermis of Tg(K6b-E6/E7) mice is not affected and skin alterations are restricted to the hair follicle renewal cycle.^26^ This is expected because *K6b* is expressed in the companion layer of the inner root sheath of hair follicles and regulated during the renewal cycle. In contrast, upon ear punch injury, a marked increase in *K6b* and *K16* expression was observed that correlates with the increased oncogene expression that, in the ear regenerating epidermal area with well-organized basal layer correlated with the restricted presence of K6b in suprabasal layers. This is similar to the growing cervical epithelium (Figure 1c) and the stimulated growing epidermis without injury.^27, 28^ The disorganized epidermis commonly seen in the growing area of regenerating ears is possibly due to the high contribution of migratory cells from the wound borders and a delay in the reconstruction of the basal layer at early regeneration stages.^21^ In agreement with our observations, the epidermis of Tg(K10-E6/E7) mice, with expected constitutive limited oncogene expression in suprabasal layers, showed hyperplasia/hyperkeratosis and dividing cells in suprabasal layers under untreated conditions but, in contrast, a marked number of dividing cells were also increased in basal layers.^29, 30^ This latter effect might be a long-term consequence of the constant re-entering into the cell cycle of suprabasal cells (see below), since neither hyperplasia in the interfollicular epidermis under untreated conditions nor increased basal cell proliferation were observed in Tg(K6b-E6/E7) mice.

An interesting observation was the large area of coincidence of *K5* and *K6b* expression and the location of most dividing cells in the growing epithelial tissue. In particular, we found that the zone of coincidence of *K6b* and *K5* expression in the cervical epithelial tissue correlates with the area where dividing cells in suprabasal layers were present. Accordingly, extension of K5 expression to upper layers has also been observed in organotypic raft cultures transfected with HPV.^31^ Therefore, K6b+/K5+ cells in the cervix might identify those susceptible for oncogene-induced cell cycle re-entry. Although we detected the highest amount of E7 in upper suprabasal layers, it has been reported that high levels of p21/p27 make differentiated cells refractory to E7 mitogenic effects.^32^

In Tg(K6b-E6/E7) mice, longer duration of the growing phases is the likely cause of the thicker cervical epithelium during proestrus–estrus phase. This is similar to the effect of E6/E7 on hair follicles, which also show higher cycling frequency and a longer growing phase (anagen) than those of WT mice.^26^ We interpreted this phenomenon as oncogenes preventing from the entry of stem/progenitor cells of the bulge into the G0 cell cycle phase, expected for resting hair follicles (telogen). An alternative interpretation accordingto the present study is that E6/E7 drives the re-entry into the cell cycle of postmitotic K6b+ cells causing hair follicle growth from these cells instead of the regular stem cells that receive the resting signals.

### Estradiol as a growth-promoting factor

Estradiol is an endocrine factor required for the initiation of growth of the cervical–uterine tract, such that lack of ERα in pituitary is sufficient to interrupt estrous cycling and the cervical tissue remains at diestrus.^33, 34^ However, in agreement with the participation of additional factors in estrous cycle regulation, continuous administration of E2 at the dose used here did not prevent from the action of factors ending the growth phase (e.g., progesterone^12^) and cycling continued. Proestrus initiation in Tg(K6b-E6/E7) mice was completely dependent on estradiol signaling and the end of the growth phase occurred as in WT mice. This is likely due to the transient *E6/E7* expression during the estrous cycle. Exogenous E2 did not increase the frequency of estrous cycles but endogenous estradiol was required to exit from diestrus in both WT and Tg(K6b-E6/E7) mice. Therefore, the reversible interruption of carcinogenesis by raloxifene recently reported^35^ could be due to the induced estrous cycle pause at diestrus, stage at which *E6/E7* are expressed at low levels and, likely, the HPV life-cycle is halted in human patients.

An additional possible role of estradiol in the regulation of estrous cycle is the growth promoting activity during proestrus.^36, 37^ Accordingly, estradiol extended the time WT cervical tissue was at the proestrus–estrus phase. In addition, we observed that after proestrus initiation, ER inhibition caused a marked reduction in basal proliferating cells of the cervix. This same effect was noted in basal and suprabasal cells of the cervix of Tg(K6b-E6/E7) mice, revealing the cooperative effect of oncogenes and estradiol. This cooperation was confirmed by the cancerous phenotype generated in Tg(K6b-E6/E7) mice treated with E2, showed even under continuous cycling. Interestingly, similar growth effects were noted in regenerating ears of both WT and Tg(K6b-E6/E7) mice, though only a mild effect was noted in regeneration efficiency. However, estradiol does not seem to be a limiting factor in this growth promoting activity in female mice, since increasing estradiol levels did not produce a marked effect on regeneration efficiency or the number of proliferating cells. ERα is the best candidate to carry out this activity since mediates estradiol mitogenic activities *in vitro*^38^ and is expressed in the lower layers of cervix^14, 37^ and epidermis epithelia.^39^ However, an indirect effect mediated by estradiol signaling in the cervical stroma has been suggested based in the expression of *ERα*^14, 37^ and its genetic inactivation^40^ in this tissue. A definitive conclusion in this regard awaits further investigation, since the *ERα* gene inactivation strategy used to support this possibility did not discard that the gene was also deleted in endocrine organs (e.g., pituitary), consequently blocking the estrous cycle and the carcinogenic process as referred above.

### Mechanisms of regeneration in carcinogenesis

**Figure 7:**
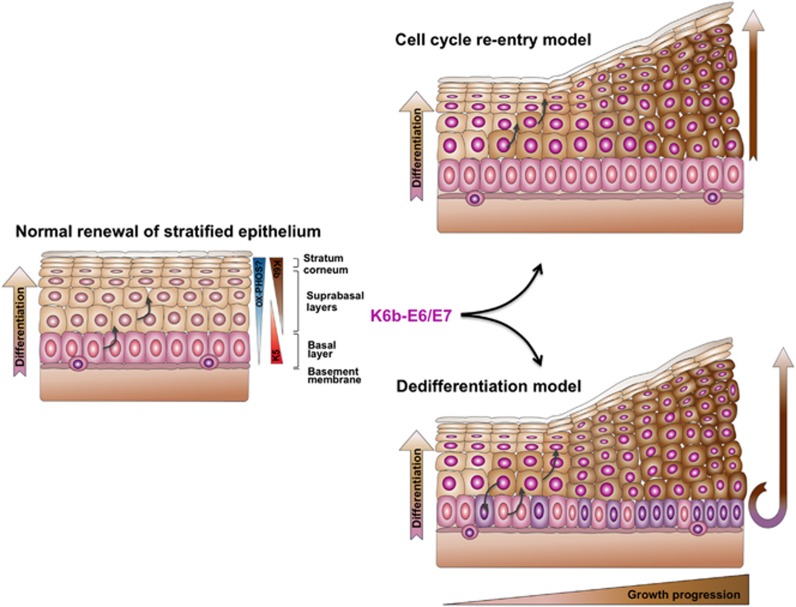
Models of suprabasal E6/E7 promoting growth effect on cervical epithelium. During normal growth (proestrus–estrus phase), the cervical stratified epithelium derives from basal stem cells that proliferate and give rise to the postmitotic cells of suprabasal layers that finally derive into the stratum corneum. In association with this process, the genes encoding K5 and K6b form opposite basal–suprabasal expression gradients, and oxidative phosphorylation could increase. As it appears to occur in HPV-infected human cervical tissue, E6/E7 oncogenes in suprabasal cells promote cell cycle re-entry directly causing increased cell proliferation and tissue growth (cell cycle re-entry model). Alternatively, possibly resulting from the re-entry into the cell cycle, suprabasal cells dedifferentiate into basal-like stem cells; growth in this case result from the increased number of basal cells (dedifferentiation model). The data presented support the first model since we were unable to detect any significant increase in basal proliferating cells due to suprabasal oncogene expression.

Postmitotic cells can contribute to tissue repair by dedifferentiation into stem cells.^41^ Our data support that the increased growth induced by HPV oncogenes at the initial stages of carcinogenesis is mostly due to the cell cycle re-entry of suprabasal cells without requiring dedifferentiation into basal-like cells (Figure 7) similar to what has been observed in regenerating tissues.^42, 43, 44^ Nonetheless, the re-entry into the cell cycle of postmitotic cells could be essential for dedifferentiation at later stages of carcinogenesis. Actually, this phenomenon might explain the increase in basal dividing cells in the epidermis of Tg(K10-E6/E7) mice as referred above. We propose that the long-term cooperation between E6/E7 and estradiol in cervical–uterine cancer development could result from the cumulative increase in new proliferating cells contributing to epithelial growth and carcinogenesis (Figure 7). In this regard, it was interesting to find an increased number of koilocyte-like and bi-nucleated cells in the chronically growing cervical epithelium of E2-treated Tg(K6b-E6/E7) mice, as occurs at the early stages of carcinogenesis in the HPV-infected human tissue.^22^

Cancer and regeneration are closely related processes. The present work supports this relationship showing that two well-known promoters of cancer, estradiol and HPV oncogenes, also promote regeneration. Furthermore, it is interesting the parallel estradiol-E6/E7 cooperation for growth observed between cervix and skin. Accordingly, as in the cervix, ERα has been implicated in skin carcinogenesis.^45^ Since ear regeneration is a simple experimental model, the data presented encourage further studies in regenerating ears of Tg(K6b-E6/E7) mice to understand the early stages in cervical carcinogenesis.

## Materials and Methods

### Animal handling and treatments

The genetic background of Tg(K6b-E6/E7)-M8 transgenic mice (always hemizygous for the transgene) was that of CD1 outbred or Fvb/N inbred strains. These and the corresponding WT mice were maintained and treated in accordance with the regulations of the local Bioethical Committee. Genotype was determined by E7-specific PCR, as previously reported.^26^ Bilateral ovariectomy was performed at the moment of ear perforation, whereas castration in males was performed 2 weeks before ear punching. Pellets embedded with 0.05 mg ß-estradiol (E2) 17-acetate (90-day release; NE-271; Innovative Research of America, Sarasota, FL, USA) were implanted in the dorsal-anterior area of mice at the day of ear perforation for long-term ear regeneration experiments; whereas, pellets embedded with 0.25 mg E2 17-acetate (90-day release; NE-271) were implanted for estrous cycle determination (19 days) or for cancer experiments (up to 3 pellets for 9 months). For short-term regeneration studies, E2 (0.7 μg/100 μl dissolved in corn oil) or the vehicle were injected subcutaneously every day up to for 14 days; hormone was not injected on the day of ear perforation or of BrdU injection. For ER inhibition, female mice were intraperitoneally injected with raloxifene (Eli Lilly, Indianapolis, IN, USA; 10 mg/ml in phosphate-buffered saline (PBS)); for regeneration experiments, 1.5 mg of raloxifene was delivered daily for 14 days or 5 days/week for a month and, during the estrous cycle, the same amount was delivered daily through 19 days or once at proestrus. BrdU (50 μg/g weight of mice) was intraperitoneally injected (1 dose) and mice killed 2 h later.

### Hole closure assay

Excisional 2 mm punches were made with a metal ear puncher on the center of ears of 6 weeks and 3-month-old mice. The hole diameter was measured 4 weeks after punching under a dissecting microscope. Formation of new hair follicles and cartilage was determined by visualizing them in samples stained with fast green and safranin after 24 h or 3 months of ear perforation.^21^ Ear regeneration efficiency was classified into four categories (gray scale in bar graphs) based on the following estimation:

Percent of regeneration (*P*_r_)=100−(100·non-regenerated area/area excised)


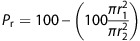


where *r*_1_=measured diameter (mm)/2, and *r*_2_ =2 mm/2=1 mm

Therefore,


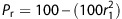


### Analysis of estrous cycle

Mice for analysis of estrous cycles were CD1-based 9 weeks old, whereas those for promotion of cervical–uterine carcinomas were Fvb/N-based 1-month-old. The estrous cycle phase was determined by analysis of cell composition and morphology of vaginal smears, taken every day at the same hour, stained with hematoxylin–eosin. The number of estrous cycles was determined by counting the number of proestrus–estrus (P–E) sequence along 19 days.^46^ Samples for molecular analysis were obtained along the estrus cycle or at specific estrus phase in the case of E2-treated mice.

### Histochemistry and immunodetection procedures

Ears and/or cervix were dissected from mice treated as described above, fixed in 4% paraformaldehyde and kept in 30% sucrose at 4 °C until sectioning. Frozen sections (10 μm) were used for all determinations. Ki67 and incorporated BrdU were the antigens used to determine the number of proliferating cells in at least six sections per sample initially subjected to a permeabilizing solution (1% Tritón X-100, 3% H_2_O_2_ in TBS) at room temperature for 15 min, then incubated with 1 N HCl for 20–30 min and neutralized with 0.1 M boric acid pH 8.5; for BrdU detection, we additionally incubated with 0.001% trypsin. All tissue sections were subjected to heat-induced epitope retrieval with ImmunoDNA Retriever Citrate (Bio SB Inc, Santa Barbara, CA, USA) at 60 °C for 30 min and treated with 0.3% H_2_O_2_ in methanol for 30 min. Tissues for keratin 5 (K5) and 6b (K6b) and E7 oncogene detection were permeabilized at this stage with 0.1% Triton X-100 in PBS for 30 min. After washing with PBS, samples were treated for 30–60 min with a blocking solution (10% mouse serum in PBS for K5, K6b and E7, and 10% donkey serum, 0.1% Triton X-100 in TBS for Ki67 and BrdU). Slides were then incubated in a humid chamber for 30–60 min at 25 °C and overnight at 4 °C with the specific antibodies (Supplementary Table S1), washed twice with PBS and incubated with the corresponding secondary antibody (Supplementary Table S1) for 2 h. Regardless of the secondary antibody used for histochemical determinations, after washing with PBS, tissue sections were incubated with the ABC Elite (Avidin/Biotin) System (1:200 dilution; Vector Laboratories, Burlingame, CA, USA) at 25 °C for 30 min. The horseradish peroxidase activity was developed using H_2_O_2_ and 3,3-diaminobenzidine. Dying cells were detected with *In Situ* Cell Detection Kit (Roche, Mannheim, Germany). Finally, tissue sections were counterstained with DAPI or hematoxylin, mounted with ProLon Gold (ThermoFisher Scientific, Waltham, MA, USA) and photographed using an invert microscope (confocal LSM 510, Apotome Axio Observer Z1, Zeiss, Jena, Germany; confocal FV1000 or BX51, Olympus, Tokyo, Japan). To count Ki67+ and BrdU+ cells, a section of tissue was taken and positive nuclei counted along a defined linear extension (at least 400 μm) of epithelium.

### Reverse transcription quantitative PCR (RT-qPCR) procedure

RNA was obtained from cervix and ears of WT or Tg(K6b-E/6E7) mice using the Hybrid-R kit (GeneAll, Seoul, South Korea). For estrous cycle analysis, 9-week-old CD1 mice were selected at each estrous cycle phase and the whole cervix dissected. For ear regeneration, 3–4 perforations were done in one ear of 3-month-old CD1 female mice and, 7 days later, both injured and non-injured ears were collected. First-strand cDNA was synthetized using HyperScript Reverse Transcriptase (GeneAll, Seoul, South Korea) and random primers (Invitrogen, Carlsbad, CA, USA). The real-time quantitative PCR was performed using KAPA SYBR FAST mix (KAPA Biosystems, Wilmington, MA, USA) in the presence of specific primers (Supplementary Table S2) and the Rotor-Gene Q (Qiagen, Wilmington, MA, USA). Gene expression was evaluated using a ΔΔCt method. The housekeeping gene *Rplp0* was used to normalize gene expression levels.

### Statistic analysis

Animals were sorted only by genotype or treatment, and although exclusion or inclusion of an animal was not predetermined, some mice of the Tg(K6b-E6/E7) died during the experimental treatment (the majority died before 2 months of age^26^); it is important to mention, however, that the cause of death was never associated with the presence of tumors. Specific blinding or randomization method was not applied. The size of each experimental group was limited according with reproducibility and extent of difference; generally, small groups (3–4 independent individuals) were only considered for determination with an evident qualitative difference between groups. Fisher's exact test was used for the analysis of ear regeneration data; since there is a low correlation between a mouse and the regeneration efficiency of its ears, each ear was taken as an independent regeneration event. For other experiments, the *t*-Student test was performed when the distribution was normal, or the Mann–Whitney Rank-Sum Test when the data distribution was not normal. Since the intact and perforated ear sample for gene expression analysis derived from the same mouse, the paired *t*-Student test was used in this case. The results are shown as the mean±s.d. and the variance analysis was performed when two data sets were analyzed. We considered significant differences when the *P*-value was ≤0.05.
