# Supplementary figures and images for: E6/E7 oncogenes in epithelial suprabasal layers and estradiol promote cervical growth and ear regeneration
Source: Oncogenesis. 2017 Aug 28;6(8):e374–. doi: 10.1038/oncsis.2017.73 (PMC5608921; doi:10.1038/oncsis.2017.73)

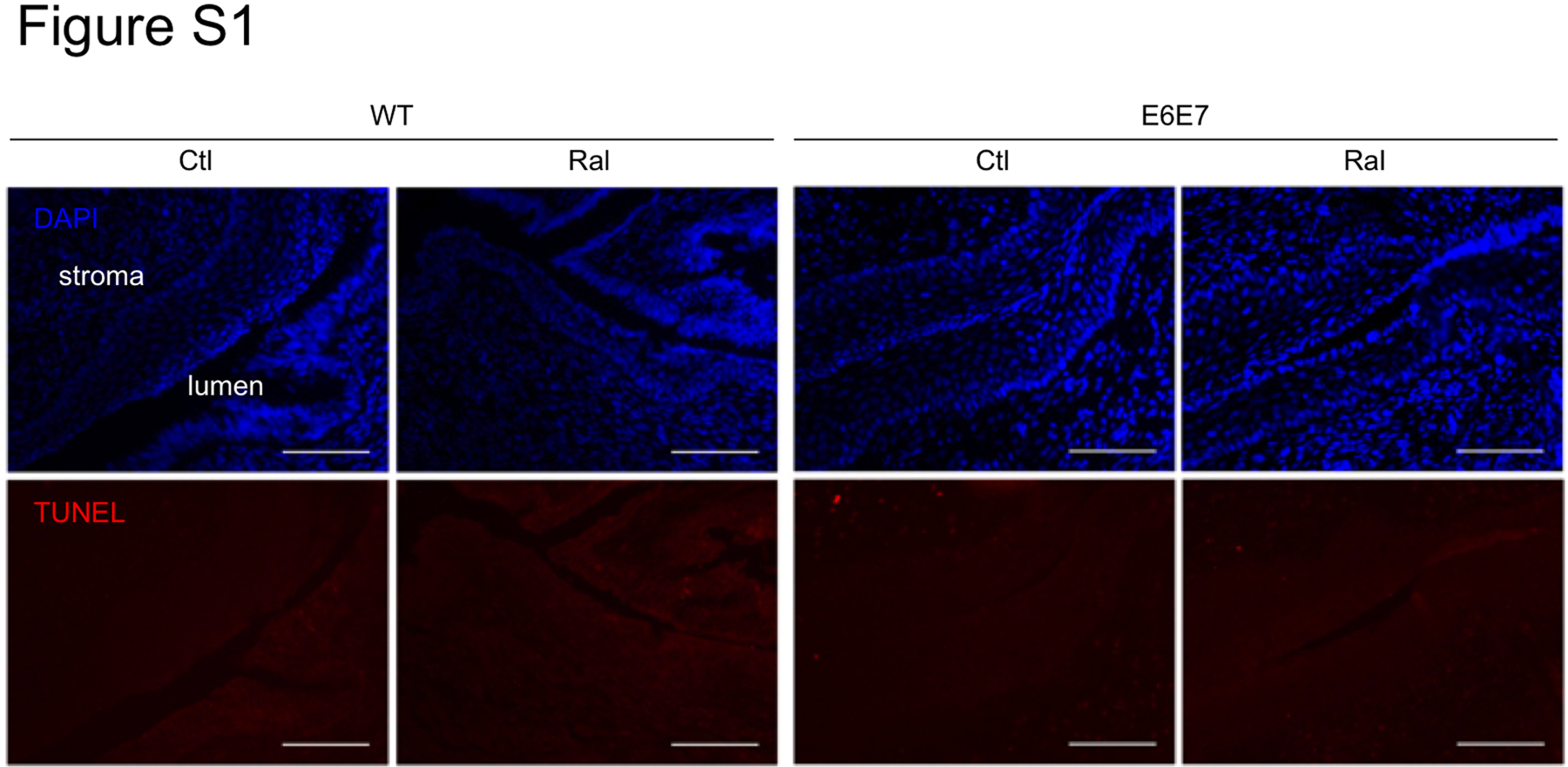

Supplement: Supplementary Figure 1 [file oncsis201773x1.tif]

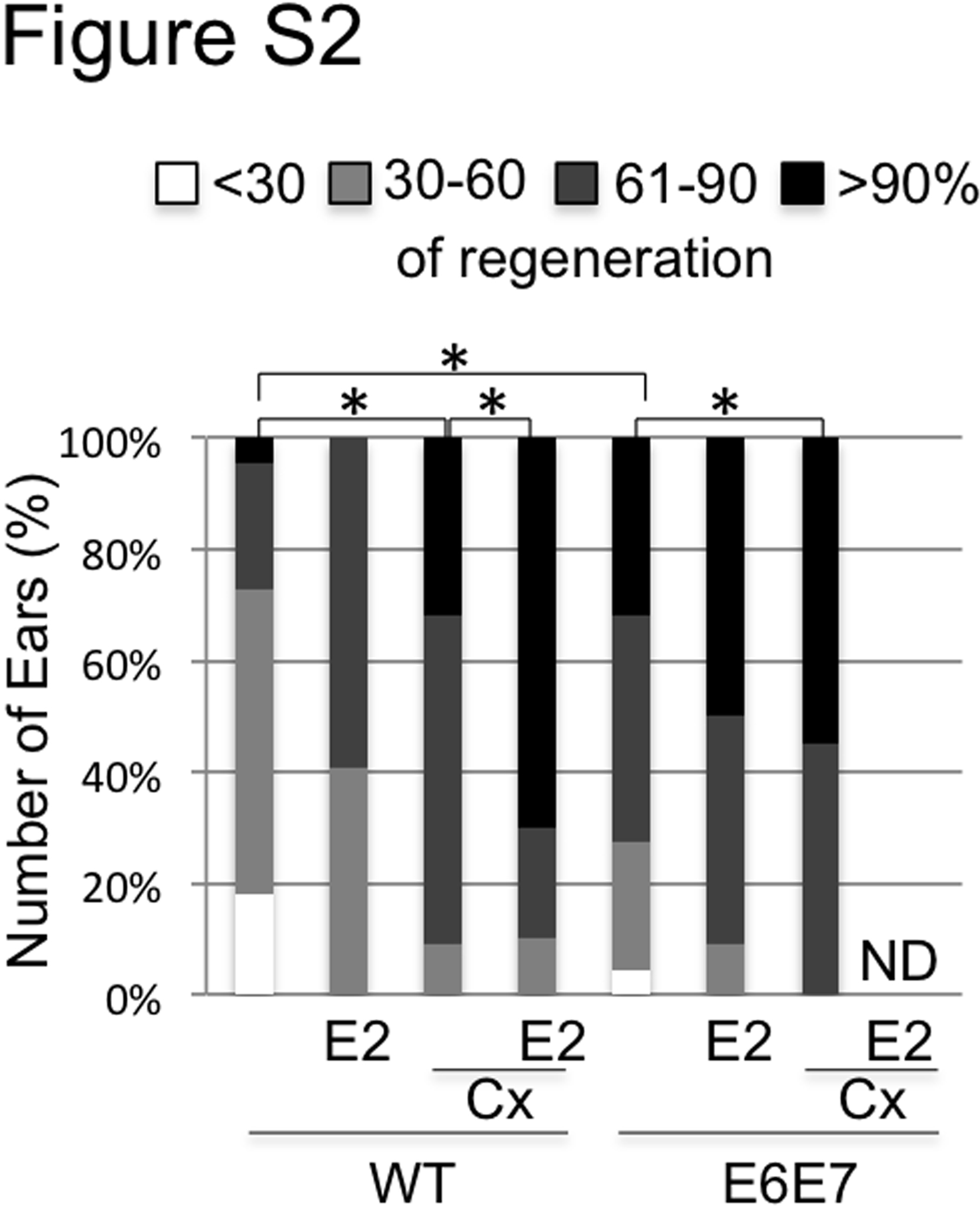

Supplement: Supplementary Figure 2 [file oncsis201773x2.tif]

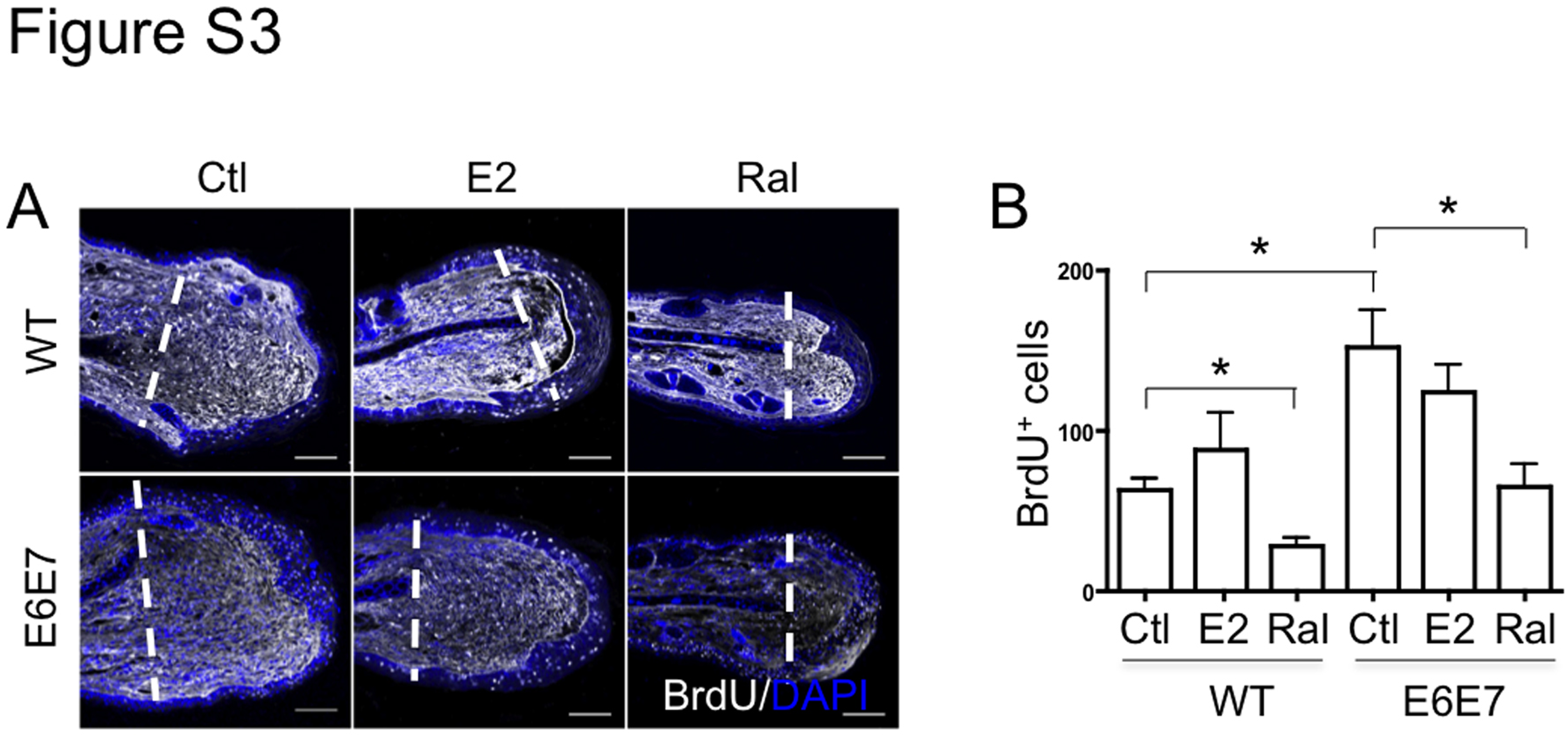

Supplement: Supplementary Figure 3 [file oncsis201773x3.tif]

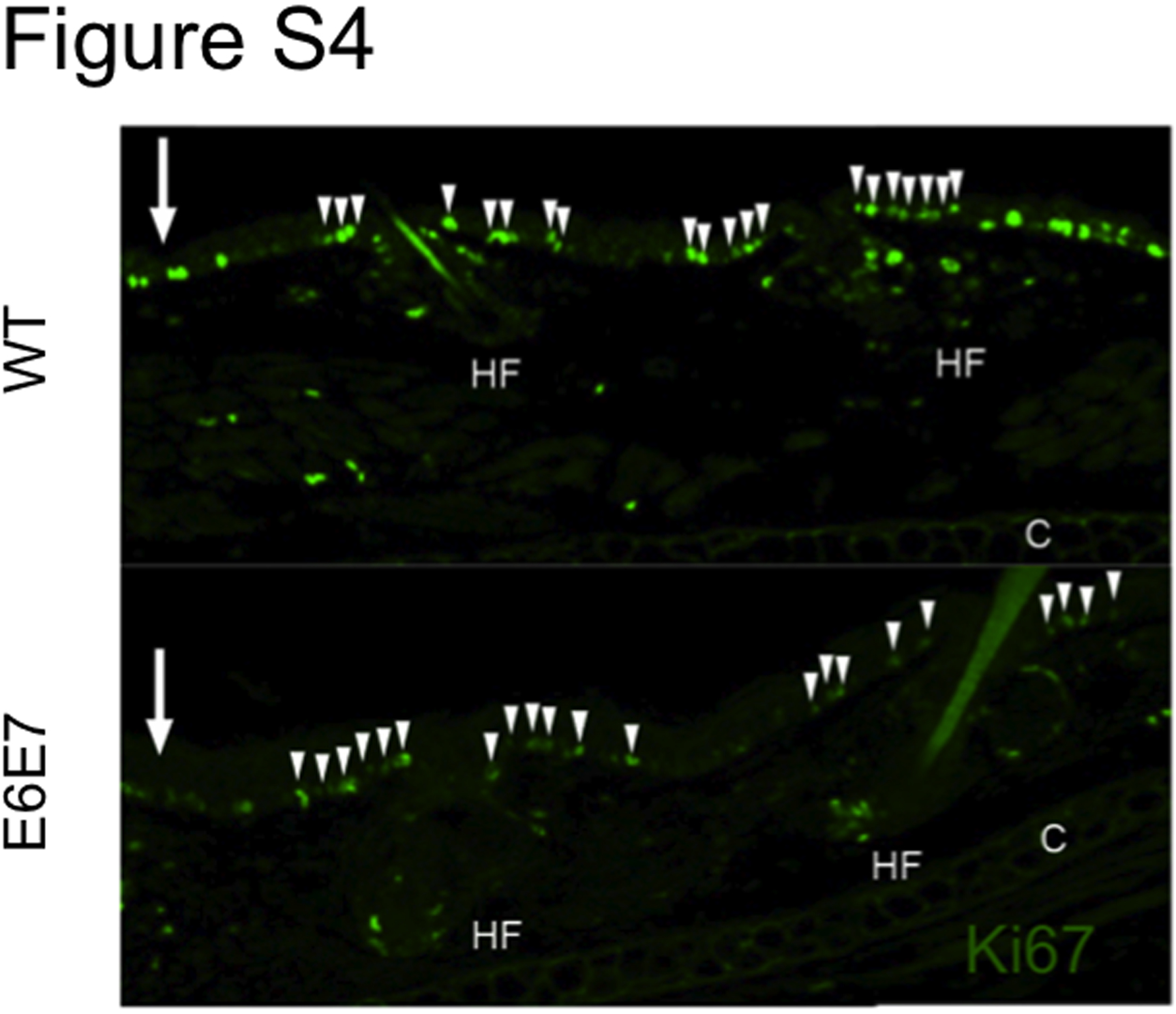

Supplement: Supplementary Figure 4 [file oncsis201773x4.tif]
