# Supplementary material for: E6/E7 oncogenes in epithelial suprabasal layers and estradiol promote cervical growth and ear regeneration
Source: Oncogenesis. 2017 Aug 28;6(8):e374–. doi: 10.1038/oncsis.2017.73 (PMC5608921; doi:10.1038/oncsis.2017.73)
Supplement: Supplementary Figure Legends [file oncsis201773x5.docx]

**Supplementary Figures Legends**

**Figure S1. Raloxifene does not induce apoptosis in the cervix epithelium.** Death cells were detected by the TUNEL technique. No evident positive cells were found in the transformation zone of the cervical epithelium of samples from treated (Ral) or untreated (PBS) mice (WT, n=3; Tg(K6b-E6/E7), n=4). Control samples were tissues treated with DNAase (not shown). Scale bar, 100 μm.

**Figure S2. Estradiol promotes ear regeneration in males.** WT and Tg(K6b-E6/E7) juvenile (6-weeks old; n=22) male mice were treated with E2 and/or castrated (Cx; n=22); ear punch was done two weeks after castration. In contrast with females, E2 in males increased the efficiency of ear hole closure. Efficiency was even higher in Cx mice and further increased when E2 treatment was done on these mice (n=10). ND, not determined. *, p<0.05.

**Figure S3. Estradiol and E6/E7 oncogenes promote BrdU incorporation during ear regeneration.** E2 or raloxifene were administered to WT (n=3) and Tg(K6b-E6/E7) (n=3) mice at the start of ear regeneration as described in Materials and Methods, whereas BrdU was injected twice 7 days after ear punching and BrdU+ cells analyzed in regenerating ears 72 h later. The majority of BrdU+ cells were located in the regenerating area (right side of dashed line) and, as expected due to differentiation, few of those were in the basal layer (A). In agreement with the number of Ki67+ cells determined (Fig. 5), a higher number of BrdU+ cells was determined in Tg(K6b-E6/E7) in comparison with WT and, although additional estradiol (E2) did not increase this number, fewer BrdU+ cells were detected in regenerating ears of mice treated with raloxifene (Ral) (B). *, p<0.05; scale bar, 100 µm.

**Figure S4. Contribution of proliferating hair follicle cells to ear regeneration.** Near the growing area (100-200 µm left from arrow), many Ki67+ cells (arrowheads) were found surrounding some hair follicles (HF) in both WT and Tg(K6b-E6/E7). The low frequency of these events does not support hair follicles as a major source of cells for ear regeneration. C, cartilage.
