# Supplementary material for: E6/E7 oncogenes in epithelial suprabasal layers and estradiol promote cervical growth and ear regeneration
Source: Oncogenesis. 2017 Aug 28;6(8):e374–. doi: 10.1038/oncsis.2017.73 (PMC5608921; doi:10.1038/oncsis.2017.73)
Supplement: Supplementary Tables [file oncsis201773x6.docx]

**SUPPLEMENTARY TABLES**

**Table S1. Antibody list.**

| **Antibody** | **Dilution** | **Company Provider** |
| --- | --- | --- |
| Cytokeratin 6b (K6b) polyclonal | 1:1000 | GeneTex  GTX110426 |
| Cytokeratin 5 (K5) monoclonal | 1:100 | Abcam (EP1601Y)  ab52635 |
| Anti-HPV E7  monoclonal | 1:100 | Santa Cruz (TVG710Y and ED17)  sc-264 and sc-6981 |
| Ki67  monoclonal | 1:50 | ThermoFisher Scientific (SP6)  MA5-14520 |
| BrdU  monoclonal | 1:50 | BD BioSciences (B44)  347580 |
| Anti-rabbit biotinylated | 1:300 | Vector Laboratories  PK-6101 |
| Anti-mouse biotinylated | 1:50 | DAKO  E0464 |
| Anti-rabbit AF 488 polyclonal | 1:1000 | Molecular Probes  A11008 |
| Anti-mouse AF 488 polyclonal | 1:1000 | Molecular Probes  A11001 |
| Anti-rabbit Alexa 594 polyclonal | 1:500 | Molecular Probes  A11012 |

**Table S2. PCR Primer list.**

| **Gen** | **Forward Primer** | **Reverse Primer** |
| --- | --- | --- |
| *K6b* | TGCTGCCTACATGACCAAGG | GAGACAGTTCTGCCTCATAAATAAC |
| *K16* | ATGGCGAGAATATCCACTCCTC | CTCCTTGAGGATGGACCGGG |
| *K14* | TCCAGAGATGTGACCTCCACC | GGGACAATACAGGGGCTCTTC |
| *K10* | CCAATCATCTAAAGGACCAAGATAC | TTCCACACGTCTGTCCAGTG |
| *K5* | CCAGTCAACATCTCTGTCGTTACA | GCCAAAGCCACTGCCAACAC |
| *E7* | ACAAGCAGAACCGGACAGAG | GCCCATTAACAGGTCTTCCA |
